# Using social values in the prioritization of research: Quantitative examples and generalizations

**DOI:** 10.1002/ece3.8394

**Published:** 2021-12-09

**Authors:** Matthew R. Falcy

**Affiliations:** ^1^ U.S. Geological Survey Idaho Cooperative Fish and Wildlife Research Unit Moscow Idaho USA

**Keywords:** decision theory, elasticity, harvest, human dimension, matrix model, social values, value of information

## Abstract

1. Identifying critical uncertainties about ecological systems can help prioritize research efforts intended to inform management decisions. However, exclusively focusing on the ecological system neglects the objectives of natural resource managers and the associated social values tied to risks and rewards of actions.
2. I demonstrate how to prioritize research efforts for a harvested population by applying expected value of perfect information (EVPI) to harvest decisions made with a density‐independent matrix population model. Research priorities identified by EVPI diverge from priorities identified by matrix elasticity analyses that ignore social utility.
3. Using a density‐dependent harvest model, the value of information about the intrinsic productivity of a population is shown to be sensitive to the socially determined penalty for implementing a harvest rate that deviates from the goal because of imperfection in estimation.
4. *Synthesis and applications*. The effect of including social values into harvest decision‐making depends on the assumed population model, uncertainty in population vital rates, and the particular form of the utility function used to represent risk/reward of harvest. EVPI analyses that include perceived utility of different outcomes can be used by managers seeking to optimize monitoring and research spending. Collaboration between applied ecologists and social scientists that quantitatively measure peoples' values is needed in many structured decision‐making processes.

## Graphical abstract

Decisions about natural resource management can be informed by ecological science and human perception of risks and rewards. This paper describes analytical methods for making decisions about animal harvest that integrate ecological and social science.

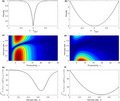

## 1 INTRODUCTION

In natural resource management contexts, good decision making includes stakeholder perceptions of the trade‐offs between conservation risks and utilization rewards. Indeed, clear articulation of objectives is necessary for good decision making (Keeney, 1992). Nonetheless, many applied ecologists exclusively focus on understanding ecological systems. While reducing system uncertainty is often needed, it may not be sufficient to improve decision making. If decisions about natural resources neglect peoples' values, then otherwise relevant ecological science can seem aloof, and the decision‐making process may appear arbitrary to stakeholders. The resulting void is filled with calls for greater integration of people into environmental decisions that are often vague and disconnected from established quantitative decision‐theoretic tools (e.g., translational ecology, Enquist et al., 2017). There is broad recognition of the need for better integration of human dimensions into natural resource management, but quantitatively synthesizing ecological science, human perceptions, and decision‐making remains challenging.

Management of harvested populations exemplifies a social trade‐off between risk and reward. There is an obvious desire to harvest as much as possible provided that current harvest does not jeopardize future harvest. Framed this way, exploitation is purely an ecological question. A quantitative ecologist armed with a matrix population model could use elasticity analysis to “Design sampling procedures that focus on estimating the vital rates where accuracy matters most” (Caswell, 2001, p. 207). Matrix elasticity analysis addresses the decision of where to direct monitoring and research efforts by focusing exclusively on the ecological system (population growth rate). How can we incorporate socially determined values about the risks and rewards of utilization and conservation into decision making? How do research and monitoring efforts to estimate population vital rates that “matter most” change if we include socially determined values about harvest?

These questions can be answered analytically by applying the expected value of perfect information (EVPI, described below) to a matrix population model. Three algebraic functions are used to model different socially determined risk/reward trade‐offs of promulgating distinct harvest rates under distinct population growth rates. Monitoring and research prioritization resulting from this analysis are compared to analogous results obtained from matrix elasticity analysis that focuses exclusively on the ecological system (population growth rate) and ignores the socially determined risk/reward trade‐off of harvest.

A comparison between EVPI and matrix elasticity isolates the effect of social values on research prioritization but uses a model of density‐independent population regulation. A second analysis applies EVPI to harvest decisions about density‐regulated populations. Analysis of the density‐dependent model will reveal the effect of socially determined penalties for missing harvest goals on the value of precisely estimating a population demographic parameter.

Analyses of EVPI are often conducted for discrete‐valued parameters, yet demographic parameters are often continuous. Implementing EVPI analyses on continuous‐valued parameters can leverage integral calculus. Here, the calculus of EVPI and the quantification of importance of social values are broadly elaborated. Population models and social risk/reward functions are generalized so that the importance of social values on research prioritization can be assessed without distraction by empirical caveats. The models described here may facilitate communication among natural resource managers, social scientists, and applied ecologists about the need, value, and methods of quantitative decision analyses.

## 2 EXPECTED VALUE OF PERFECT INFORMATION

The expected value of perfect information (EVPI, Raiffa & Schlaifer, 1961) quantifies the benefit from resolving uncertainty prior to making a decision. It uses the perceived benefits/costs associated with taking alternative actions under alternate states of reality and returns the value reaped from correctly assessing reality over some baseline of uncertainty. EVPI can be used to prioritize research and monitoring around the uncertainties that “matter most,” where “mattering” is defined in terms of the utility of actions. In applied ecological contexts, EVPI has been used to (1) design monitoring programs that address stakeholder conservation concerns (Runge et al., 2011), (2) identify the switch‐point between monitoring and acting (Bennett et al., 2018), (3) spatially prioritize conservation efforts (Raymond et al., 2020), and (4) quantify the species‐persistence benefits of reducing the most important uncertainty‐species responses to threat alleviation (Nicol et al., 2019). EVPI has also been focus of reviews (Bolam et al., 2019; Canessa et al., 2015), and analytical methods also accommodate imperfect information (Nicol et al., 2019; Raiffa & Schlaifer, 1961; Williams & Johnson, 2015).

Formally, the expected value of perfect information is

(1) $$\text{EVPI}=\int \left[\max_{\psi \in \Psi }u\left(\psi ,\theta \right)\right]f\left(\theta \right)d\theta ‐\max_{\psi \in \Psi }\left[\int u\left(\psi ,\theta \right)f\left(\theta \right)d\theta \right],$$

where $u\left(\psi ,\theta \right)$ is the utility of taking action ψ given state parameter *θ*. Utility is a measure of the total satisfaction received from a given outcome. For example, utility could be the amount of money people are willing to pay for a given level of harvest or population viability. The first square bracket is the maximum utility over all possible actions given the state parameter. Multiplying this into the probability of the state parameter taking on a given value, $f\left(\theta \right)$, and then integrating across all possible state parameter values yields the expected utility assuming rational actions for the given state. The second term subtracts off the utility obtained from taking actions that give maximum utility across all parameter states. Thus, EVPI is the value obtained from making rational decisions under perfect information about state parameters minus the value obtained from making rational decision that are constrained by a baseline of uncertainty about potential values of the state parameter. The difference (EVPI) quantifies what can be gained by switching from rational evaluation of potential states under current uncertainty to perfect knowledge of state.

## 3 METHODS

### 3.1 Matrix model

Steelhead (*Oncorhynchus mykiss*) are anadromous; they breed in freshwater and rear in the ocean. Many steelhead populations are composed of individuals that return from the ocean between ages 3 through 6 to breed in freshwater. Most individuals die after their first breeding event (semelparity) but some will make a second trip to the ocean and back to freshwater to breed again (iteroparity). A population transition matrix, *A*, for such steelhead that includes freshwater harvest of adults prior to breeding is

$$A=\left[\begin{array}{cccccc} 0 & 0 & s_{1}b_{3}\left(1‐h_{3}\right)f_{3}/2 & s_{1}b_{4}\left(1‐h_{4}\right)f_{4}/2 & s_{1}b_{5}\left(1‐h_{5}\right)f_{5}/2 & s_{1}b_{6}\left(1‐h_{6}\right)f_{6}/2\\ s_{2} & 0 & 0 & 0 & 0 & 0\\ 0 & s_{3} & 0 & 0 & 0 & 0\\ 0 & 0 & \left(1‐b_{3}\right)s_{4}+b_{3}\left(1‐h_{3}\right)r_{3}z_{4} & 0 & 0 & 0\\ 0 & 0 & 0 & \left(1‐b_{4}\right)s_{5}+\left(1‐r_{3}\right)r_{4}b_{4}\left(1‐h_{4}\right)z_{5} & 0 & 0\\ 0 & 0 & 0 & 0 & \left(1‐b_{5}\right)s_{6}+b_{5}\left(1‐h_{5}\right)\left(1‐r_{4}\right)r_{5}z_{6} & 0 \end{array}\right]\;,$$

where *s* is survival probability, b is breeding probability, *h* is harvest rate, f is fecundity in terms of eggs per female, r is repeat breeding (iteroparity) probability, z is survival of individuals attempting to breed a second time, and subscripts give the postbreeding age of individuals. For 3 year old steelhead to produce 1 year old offspring, the parent must return to breed as a soon‐to‐be 3 year old ($b_{3}$), not be harvested (1−$h_{3}$), deposit eggs ($f_{3}$; division by 2 for 50:50 sex ratio), and the eggs must survive to age 1 ($s_{1}$). There are two ways a 3 year old fish becomes a 4‐year‐old fish. It may not return to freshwater to breed (1−$b_{3}$) and then survive its fourth year ($s_{4}$), or it may return to freshwater to breed as 3 year old ($b_{3}$), avoid harvest (1−$h_{3}$), attempt to breed the following year (iteroparity, $r_{3}$), and successfully survive ($z_{4}$). Survival of older fish follows a similar pattern except that r_t+1_ is discounted by the quantity (−$r_{t}$) in order to enact a population‐level correction such that steelhead attempting iteroparity cannot have previously attempted iteroparity. All state parameter values used in matrix *A* are given in Table 1. Note that the maximum age is 6 because all 5‐year‐old fish must return to breed the following year ($b_{6}$ = 1). According to the matrix, all such fish die after spawning, and there is no possibility of becoming 7 years old.

**TABLE 1:** Parameter values of the population projection matrix *A* (top). Variance and standard deviation used for scenarios of low and high certainty (square brackets) in calculations of expected value of perfect information (bottom)

Parameter	Age 1	Age 2	Age 3	Age 4	Age 5	Age 6
*s*	0.02	0.2	0.8	0.8	0.8	0.8
*b*			0.4	0.5	0.9	1
*f*			2000	2500	3000	3000
*r*			0.4	0.2	0.2	0
*z*				0.2	0.2	0.2
$\sigma_{s}^{2}$	[0.01, 0.02]	[0.05, 0.1]	[0.05, 0.1]	[0.05, 0.1]	[0.05, 0.1]	[0.05, 0.1]
$\sigma_{f}$			[200, 500]	[200, 500]	[200, 500]	[200, 500]

The transition matrix *A* implies a density‐independent population growth rate, *λ*, which is the dominant real eigenvalue of *A*. Because decisions about harvest rates, *h*, should be predicated on the magnitude of *λ*, it is prudent to ask which matrix entries have the largest effects on *λ*. These are the life history events that need to be well estimated, and thus seemingly deserve research and monitoring priority (Caswell, 2001, p. 207). Elasticity analysis yields the proportional sensitivity in *λ* relative to proportional change in the transition matrix cell entries, *α_ij_*. Matrix *A* contains many *α_ij_* that are defined by several parameters. It is possible to perform the elasticity analysis in terms of these lower‐level parameters. Decomposing the elasticity analysis into constituent parameters *s*, *b*, *h*, *f*, *r*, and *z* provides greater resolution into important population processes. Let *x* represent any of the constituent parameters within cell *α_ij_*. The elasticity of population growth rate, *λ*, to a lower‐level parameter is

$$\frac{x}{\lambda }\frac{\partial \lambda }{\partial x}=\frac{x}{\lambda }\sum_{\mathrm{i,j}}\frac{\partial \lambda }{\partial \alpha_{\mathrm{ij}}}\frac{\alpha_{\mathrm{ij}}}{\partial x}.$$

The first term inside the summation is the sensitivity of *λ* to a given projection matrix cell entry, *α_ij_*. These sensitivities are then multiplied into the partial derivative of *α_ij_* with respect to the constituent parameter *x*, summed across all cells and then scaled by the magnitude of *x* relative to *λ*. Calculating the elasticity of *λ* with respect to *b*_3_ thus begins by finding the partial derivative of *λ* with respect to *b*_3_ for cell *α*_13_

$$\frac{\partial \lambda }{\partial b_{3}}=\frac{f_{3}\left(1‐h_{3}\right)s_{1}}{2}$$

and the other cell in which *b*_3_ appears, cell *α*_43_

$$\frac{\partial \lambda }{\partial b_{3}}=z_{4}\left(r_{3}‐h_{3}r_{3}\right)‐s_{4}.$$

These partial derivatives are summed and then multiplied by the quotient, $\frac{b_{3}}{\lambda }$.

### 3.2 Incorporating social values

**FIGURE 1:**
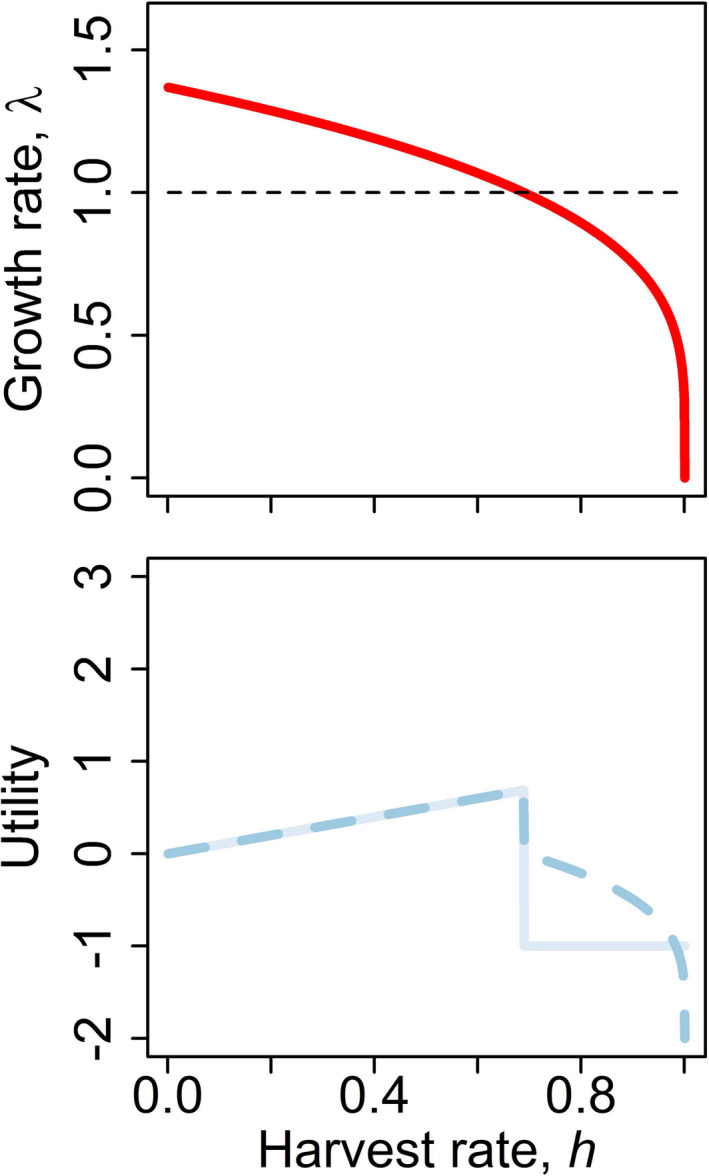
Population growth rate computed from the population transition matrix *A* parameterized with values given in Table 1 (top). Horizontal dashed line references population replacement. Three utility functions increase with harvest rate until population growth rate becomes negative (bottom)

The foregoing elasticity analysis will identify critical parameters in the ecological system. This could be used to focus research and monitoring on the most important parameters with respect to *λ*, but it neglects the objectives of managers, which are influenced by society. Managers may reap greater reward with increasing harvest rate provided that postharvest population growth rate is positive. The reward may be negative (penalty) for promulgating harvest rates that cause negative population growth. Thus, there may be a precarious motivation to harvest up to, but not exceed, rates that permit positive population growth. Three such utility functions are given below and in Figure 1.

$$\begin{array}{cc} u_{1} & \propto \left\{\begin{array}{cc} ‐1 & \text{if}\;\lambda <1\\ h, & \text{if}\;\lambda >1 \end{array}\right.\\ u_{2} & \propto \left\{\begin{array}{cc} ‐2+2\lambda , & \text{if}\;\lambda <1\\ h, & \text{if}\;\lambda >1 \end{array}\right.\\ u_{3} & \propto \left\{\begin{array}{cc} ‐4+4\lambda & \text{if}\;\lambda <1\\ 5h^{2}, & \text{if}\;\lambda >1 \end{array}\right. \end{array}$$

Each utility function *u*_1_, *u*_2_, and *u*_3_ gives the utility of harvest at level *h* (*h* is the action we can take, which can be any number on the interval [0, 1]) given the effect this action has on *λ*. Using some set of values for state parameters $\theta \equiv \left\{s\left.,\;b,\;f,\;r,z\right\}\right.$ we can calculate the utility of harvest at level *h* by doing the Eigen analysis of matrix *A* to get *λ* and then using the result to evaluate the function *u*. Thus, EVPI can be calculated for all state parameters and utility functions, regardless of whether the utility functions arise from empirical data or formal methods of judgment. Indeed, the form of the utility function depends on people's objectives, which social scientist may help to identify. A probability density function $f\left(\theta \right)$ is required to model plausible state parameter values. This is derived from the same data used to generate point estimates of the state parameters *θ*. If data do not exist, then $f\left(\theta \right)$ is a prior distribution arising from professional opinion and literature review.

### 3.3 Uncertainty and EVPI

The state parameter for survival‐at‐age, *s*, is a number on the interval [0, 1]. The beta distribution is a suitable probability density function, $f\left(\theta \right)$, to model plausible values of *s*. The beta distribution was reparameterized in terms of mean μ and variance *σ* ^2^:

$$f\left(s\right)=\;\frac{\Gamma (a)\Gamma (b)}{\Gamma (a+b)}$$

where Γ is the gamma function, Γ(X+1) = X!, and by method of moments

$$a=\;\mu \left(\frac{\mu \left(1‐\mu \right)}{\sigma^{2}}‐1\right)$$

$$b=\;\left(1‐\mu \right)\left(\frac{\mu \left(1‐\mu \right)}{\sigma^{2}}‐1\right).$$

It is thus possible to “center” $f\left(s\right)$ on values given in Table 1 while entertaining scenarios of relatively low and high certainty, *σ* ^2^. Two levels of certainty in fecundity‐at‐age, *f*, were modeled with the normal distribution, which is parametrized by mean and standard deviation (Table 1).

The harvest action ψ is one of nine rates *Ψ* = {0.1, 0.2,…, 0.9}. This discretization is likely fine‐scale relative to the degree of management control over harvest rate (Eriksen et al., 2018). For simplicity, matrix elasticity and EVPI are compared only for survival (*s*) and fecundity (*f*) state parameters.

### 3.4 Density‐dependent model

**FIGURE 2:**
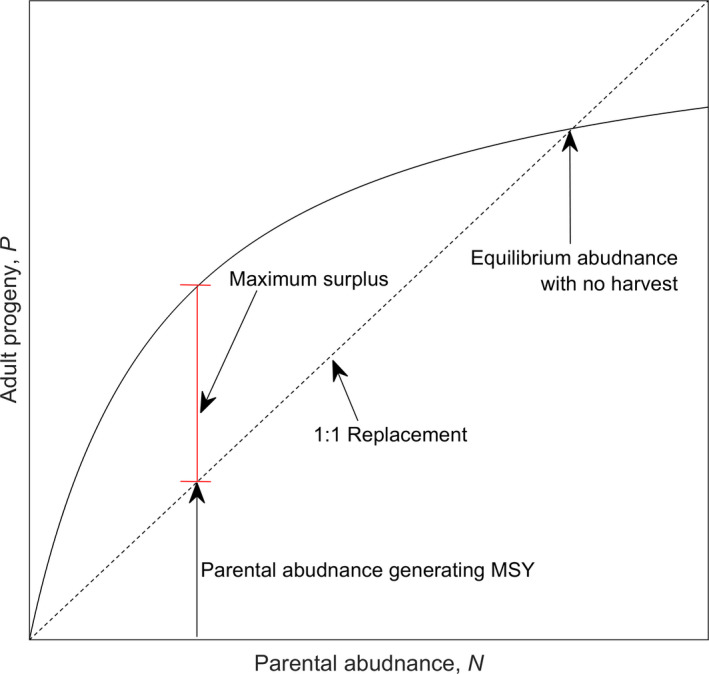
The Beverton‐Holt (1954) model of harvestable parental abundance, *N*, has a density‐dependent effect on subsequent adult progeny, *P*, available for future harvest. Maximum sustained yield (MSY) is associated with the greatest vertical distance (red) between the recruitment function (curved line) and the replacement line (dashed). Intrinsic productivity is the slope of the curve at the origin. Lognormally distributed uncertainty in intrinsic productivity translates to left‐skewed uncertainty in MSY (Appendix A)

The matrix model of section 3.1 is density‐independent. A density‐dependent recruitment model for semelparous animals was proposed by Beverton and Holt (1957)

$$P=\frac{\alpha N}{1+\frac{\alpha }{\beta }N},$$

where *P* is the abundance of adult progeny produced by *N* parents. The parameters *α* and *β* are the slope at origin (“intrinsic productivity”) and asymptote, respectively, of the recruitment model (Figure 2). The harvest rate, *h*, that gives rise to maximum sustained yield (MSY) is

$$h_{\text{MSY}}=1‐\sqrt{\frac{1}{\alpha }}.$$

**FIGURE A2:**
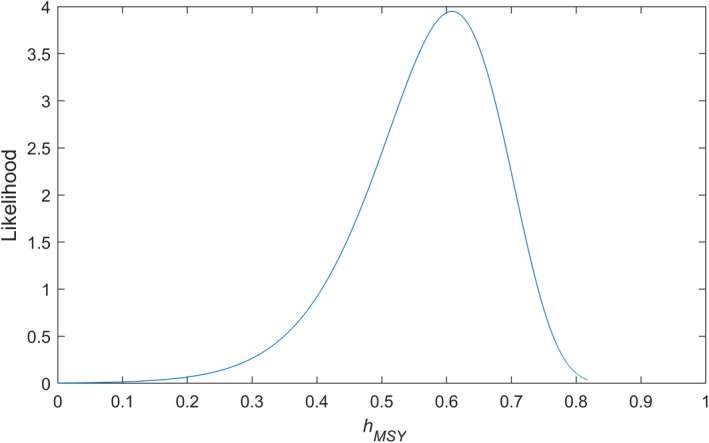
The likelihood profile of harvest rates associated with maximum sustained yield (*h*_MSY_) is derived from the transformation‐of‐variable technique, where uncertainty in intrinsic productivity is log normal with mean 1.75 and standard deviation 0.5

Intrinsic productivity, *α*, is never known perfectly; hence, there is uncertainty in *h*_MSY_. If uncertainty in intrinsic productivity, *α*, is modeled with a lognormal distribution (strictly positive) with mean 1.75 and standard deviation 0.5, then the distribution of potential values of *h*_MSY_ is skewed left (Appendix A, Figure A2). Unlike the matrix model, this model has a single biological parameter, *α*, that uniquely determines *h*_MSY_. Rather than focusing on which parameters are most important to know, this EVPI analysis will demonstrate sensitivity to the function used to model the penalty for harvesting at a rate deviating from a target that is imprecisely estimated.

**FIGURE 3:**
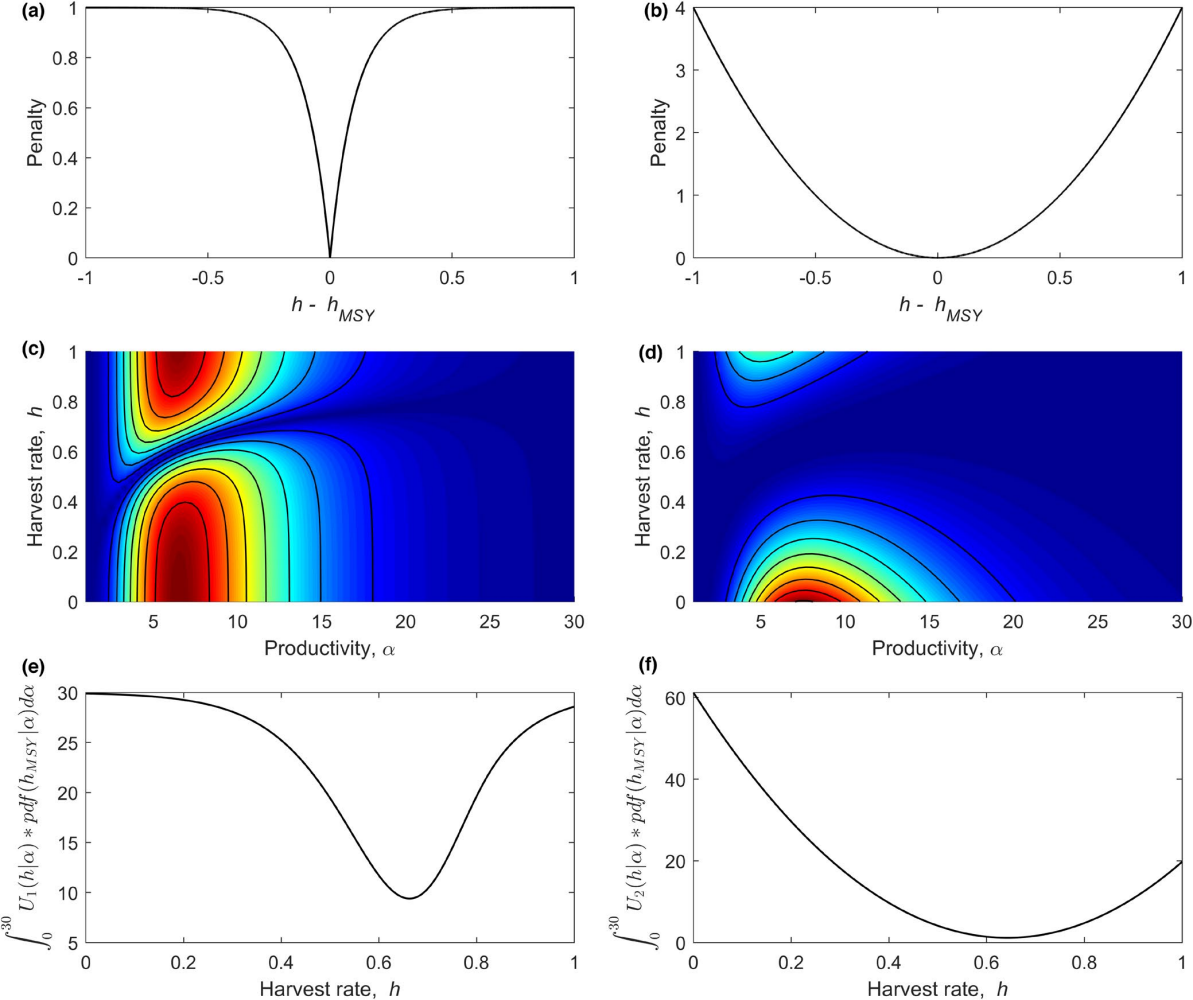
Penalties associated with different harvest rates, where the objective is to implement maximum sustained yield (a and b). Combining these penalties with lognormal uncertainty in intrinsic productivity leads to different likelihood‐weighted utilities associated with all possible combinations of intrinsic productivity, α, and harvest rate, *h*, where hotter colors represent higher penalties (c and d). The area under curves along the x‐axis of panels c and d is plotted in the y‐axis of panels (e and f) for all possible values of harvest rate. The height where the curve in e or f reaches its minimum is the expected value of perfect information. When the penalty for deviating from MSY is relatively lenient (b), the expected value of perfect information (f) is relatively low

Let the socially determined (stakeholder) objective be to promulgate a harvest rate that gives rise to MSY, where MSY is imperfectly known. Two functions for the “utility” associated with implementing harvest level *h* are

$$U_{1}\left(h\right)=1‐e^{‐10\left(\left|h_{\text{MSY}}‐h\right|\right)}$$

$$U_{2}\left(h\right)=2{\left(h_{\text{MSY}}‐h\right)}^{2},$$

which are plotted in Figure 3a and b, respectively. Defined this way, “utility” is a penalty to be minimized, which occurs when $U\left(h_{\text{MSY}}=h\right)=0$. Other utility functions not described here could more closely reflect particular stake holders’ perceptions of the risk and reward of over and under harvest. For example, bioeconomic analyses that incorporate a discount factor to future harvest benefits (Dichmont et al., 2010; Duncan et al., 2010; Grafton et al., 2010) could be incorporated through utility/penalty functions that have a more complicated relationship to MSY than those used here.

Perfect information about α (and hence *h*_MSY_) permits harvest decisions that always minimize the penalty and so the first term on the right‐hand side of Eq. 1 is 0. We may now calculate EVPI by computing only the second term on the right‐hand side of Equation (1), which is the utility (penalty) associated with making harvest decisions given the uncertainty in *α*. Computing the second term of Equation (1) can be visualized by first plotting the *α* likelihood‐weighted utility associated with all combinations of *h* and *α* (Figure 3c and d). The task is to choose *h* that minimizes the penalty over all possible levels of α. This is done by finding the horizontal slice through Figure 3c or d that encounters the most amount of dark blue. To illustrate, let two corners of a sheet of paper span the *x*‐axis of Figure 3c or d along a single value of the *y*‐axis. Pulling the sheet of paper up along the *z*‐axis (perpendicular to the *x*‐*y* plane), a line traces the intersection of the paper with the surface depicted with colors. We find the area under this line. We repeat for miniscule movements of the sheet over the *y*‐axis (Figure 3e and f). The point where the curve in Figure 3e or f reaches a minimum is the harvest rate that minimizes the likelihood‐weighted penalty (*x*‐value), which is the second term of Equation 1 (*y*‐value).

## 4 RESULTS

**FIGURE 4:**
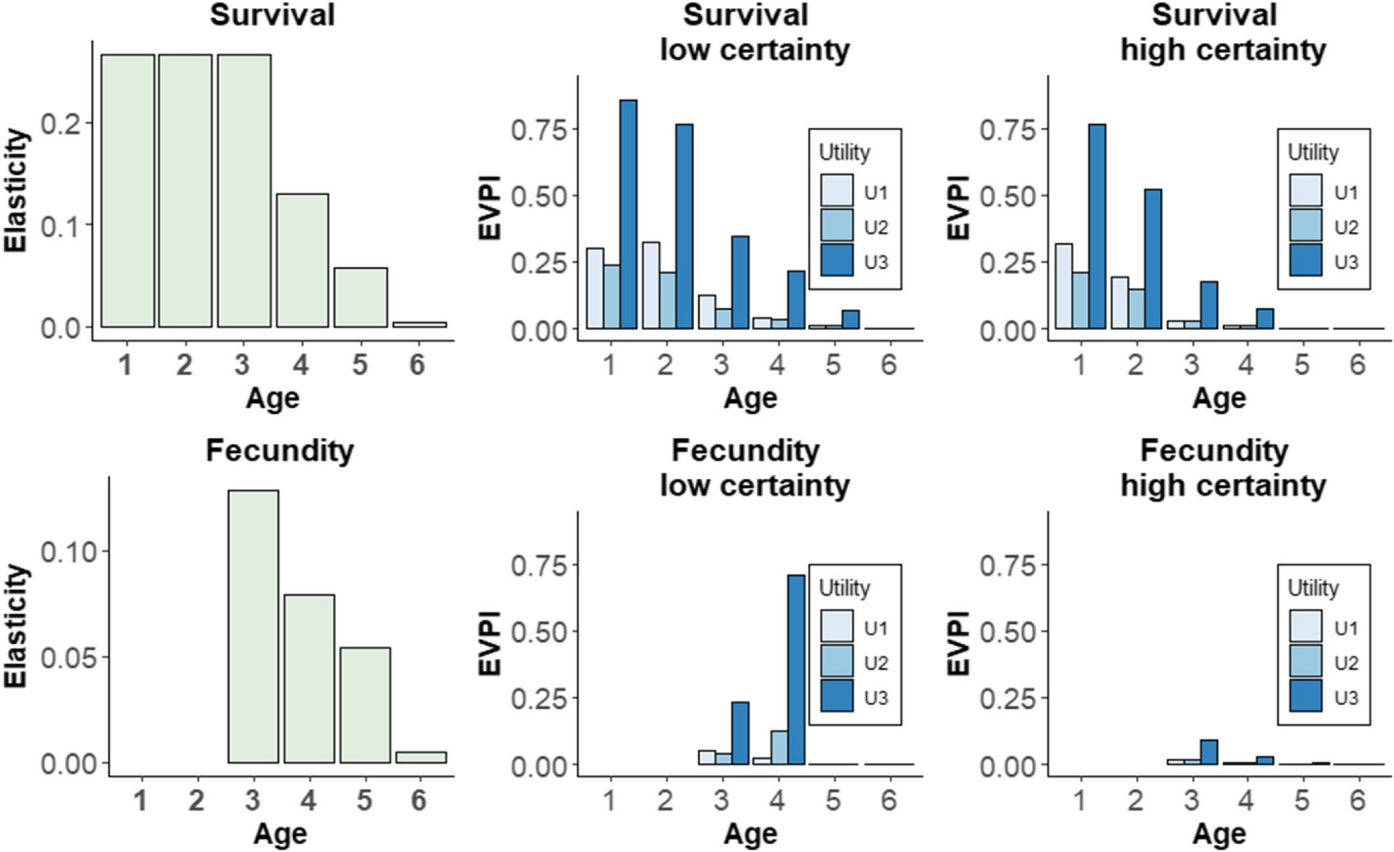
Comparison of matrix elasticity analysis (green) and expected value of perfect information analysis (EVPI, blue) for survival‐at‐age (top row) and fecundity‐at‐age (bottom row). Bar height is proportional to importance of survival or fecundity‐at‐age. EVPI panels contain results for three utility functions and two levels of uncertainty. Units of elasticity and EVPI are not directly comparable. EVPI analysis includes the effect of the socially determined utility, whereas elasticity analysis focuses exclusively on the ecological system (population growth rate)

Elasticity analysis shows that survival values to ages 1, 2, and 3 (*s*_1_, *s*_2_, *s*_3_) are equal to one another and more important to know than any other parameter (*s*_4_, *s*_5_, *s*_6_, *f*_3_, *f*_4_, *f*_5_, *f*_6_; Figure 4). However, the EVPI analysis shows that *s*_1_ is most important if the third utility function is used for both levels of certainty. EVPI analysis further shows that *s*_2_ is slightly more important than *s*_1_ if the first utility function is used and certainty is low. Increasing certainty causes this to flip so that *s*_1_ is once again most important. Both elasticity and EVPI analyses indicate declining importance of survival beyond age 3. EVPI for *s*_6_ is zero for all three utility functions under high and low certainty. More generally, increasing the prior certainty decreases EVPI, which can be deduced from first principles.

Fecundity is generally much less important than survival using elasticity analysis (note different scales on the two elasticity panels in Figure 4). The same is true for EVPI analysis, except that *f*_4_ is quite important under low certainty and the third utility function. Similarly, the elasticity analysis finds decreasing importance of fecundity with increasing age, which is also found by EVPI analysis except for the first and second utility functions under low certainty.

The function in Figure 3e reaches a minimum at 9.4. This is the value of the second term in Eq. 1. Because the first term of Eq. 1 is 0 (because of how the utility function was defined), 9.4 is the expected value of perfect information. Similarly, the function in Figure 3f reaches a minimum at 1.2. The EVPI changes across the two columns of Figure 3 because of differences in the penalty function. Rapidly increasing the penalty in the vicinity of the desired target leads to greater value of information associated with perfect estimation of the target.

## 5 DISCUSSION

Questions about harvest lead to questions about data availability, analysis, and robustness of operating models (policy) to uncertainty. This can be formalized with management strategy evaluation (Butterworth, 2007; Punt et al., 2014). Management strategy evaluation is sufficiently broad to include socially determined values, and would address the effect of resolving uncertainty using simulation (Mäntyniemi et al., 2009). Here, a purely mathematical method, as opposed to simulation, is used to compare two methods of determining critical uncertainties. Cohen et al. (2016) used bootstrap simulations to generate a distribution of matrix model parameters values and the associated λ. These were used as inputs to a subsequent EVPI analysis about a binary choice intended to maximize λ. Such analysis relies upon the bootstrap simulation to generate a distribution of discrete values that can be subsequently summed. This is distinct from the application of integral calculus to continuous‐scale parameter values used here. Furthermore, the objective of the matrix population analysis is to provide a direct comparison between EVPI(*U*(*λ*)| *f*(*θ*)) to Elasticity(*λ*|*θ*), which has not been previously explored.

The EVPI of some matrix parameters is 0 (e.g., *s*_6_, Figure 4) because the harvest decision will not change even if perfect knowledge of the parameter was available. There is no possibility for decision improvement for two related reasons. First, other parameters dominate the decision about harvest rate. For example, the elasticity of *s*_6_ is low because it occurs after survivals *s*_1_, *s*_2_, …, *s*_5_. Second, the harvest decisions were discretized into 9 levels (0.1, 0.2, …, 0.9) and so the effect of obtaining perfect knowledge of *s*_6_ is insufficient to cause a change in harvest at the Δ0.1 level. Indeed, the EVPI of *s*_6_ goes from 0 to 0.004 if the resolution of the harvest decision is increased from tenths to thousandths.

The steelhead matrix model does not address density dependence. Analyzing linear (density independent) matrix models for the maximum harvest level at which *λ* = 1 makes sense only when using low‐density vital rates (Caswell, 2001, p. 640). As stated by Caswell (2001, p. 641) “… a harvest schedule that reduces *λ* to 1 leaves the population balanced on an extinction knife‐edge. Uncertainty in parameter estimates and stochasticity (demographic or environmental) would increase the danger that a harvest policy might unintentionally drive the population to undesirably low levels.” This analysis explores the consequences of an uncertainty‐induced transgression of that knife edge, where “undesirability” is explicitly captured by the knife edge in the utility functions.

There is a rich literature on population harvest that stresses the importance of density‐dependent population regulation (Ricker, 1954; Sutherland, 2001; Walters & Maguire, 1996). Density‐dependent optimal harvest can be studied with analyses of MSY, which has a long and nuanced history (Larkin, 1977; Pauly & Froese, 2020). Managing for MSY is complicated by numerous factors. For example, an equilibrium view of population dynamics and the associated assumption of parameter stationarity are questionable (Andrewartha & Birch, 1954; Rollinson et al., 2021); mechanisms linking exogenous drivers to life history strategies are important yet difficult to know (Rose et al., 2001; Winemiller, 2005); and maximizing long‐term economic yield (Grafton et al., 2012) requires strong institutions to maintain stakeholder commitment (Dichmont et al., 2010).

Density‐dependent population regulation and associated estimates of MSY can be blended with perceived risk and rewards of harvest. Here, two functions were used to create increasing penalties as the implemented harvest rate deviates from MSY. Any alternative penalty function could be chosen based on stakeholder perceptions of the risks and rewards of harvest. For example, harvesting at level 10% above MSY may be perceived as a greater risk than harvesting at a level 10% below MSY.

The matrix model analysis demonstrates that research and monitoring priorities depend on whether the prioritization is derived from matrix elasticity analyses or EVPI analysis. Only the latter incorporates socially determined utilities representing the rewards and risks of harvest, and may be used if decision makers want to incorporate stakeholder values. The utility function provides the critical link between people and the ecological system. Because priorities can be sensitive to the form of the utility function, it is important that utility functions are appropriately formulated. Social scientists can help formulate utility functions by designing and analyzing “stated preference” studies of stakeholders (Johnston et al., 2017). Components of stated preference studies relevant to natural resource management include choice experiments and the “subjective well‐being” associated with nonmarket ecosystem services (Lindberg et al., 2020). However, these methods are not free of controversy (see Johnston et al., 2017) and cannot be known with perfection. Thus, exploring sensitivity to different utility functions requires an additional tier of consideration and analysis. The density‐dependent model demonstrated different magnitudes of value of perfect information under different formulations of the penalty function. Not surprisingly, the value of perfect information is lower when penalties for misidentifying the true target harvest rate are lower (Figure 3).

Applied ecologists can help create formal tools for translating quantitative results to decisions. The elaboration and dissemination of such tools (e.g., Conroy & Peterson, 2013) is needed to overcome the cognitive biases associated with informal decision making (Tversky & Kahneman, 1974) and implement cost‐optimizations that “do more with less” (Falcy, 2018). An impediment to robust optimization of environmental decision making is the time and expertise needed to construct appropriate models. Even the mere decision to calculate EVPI entails a human resource cost that stands outside the eventual EVPI calculus. Thus, there is a start‐up cost attached to the business of prudent decision making, and it is reasonable to ask whether this business is viable when running at different scales. Indeed, intuition is free and fast while modeling is neither. There is an emerging awareness and suspicion of human proclivity to favor free and fast intuition (Kahneman, 2011).

It should be no surprise that what people want affects what needs to be known. Quantifying the effect of including social values into decisions using rigorous analytical methods is nonetheless rare. This piece describes one small component of a much broader, structured decision‐making process for integrating people into environmental decisions (Gregory et al., 2012). Applied ecology will benefit from more examples of quantitative tools that integrate social values into decision making, lest our science seem aloof or irrelevant to the people it intends to serve.

## 6 CODE

R computer code for recreating all the matrix analyses and extending it into other state parameters is given in Appendix S1. MATLAB computer code for creating all analyses for the density‐dependent model is given in Appendix S2. MATLAB computer code supporting Appendix A is given in Appendix S3.

## AUTHOR CONTRIBUTION

**Matthew R. Falcy:** Conceptualization (lead); Formal analysis (lead); Methodology (lead); Software (lead); Writing‐original draft (lead); Writing‐review & editing (lead).

### OPEN RESEARCH BADGES

This article has earned an Open Materials Badge for making publicly available the components of the research methodology needed to reproduce the reported procedure and analysis. All materials are available at Appendix S1 includes code for replicating all analyses described in the manuscript.

## Supporting information

Appendix S1

Appendix S2

Appendix S3

## Figures and Tables

**FIGURE A1:**
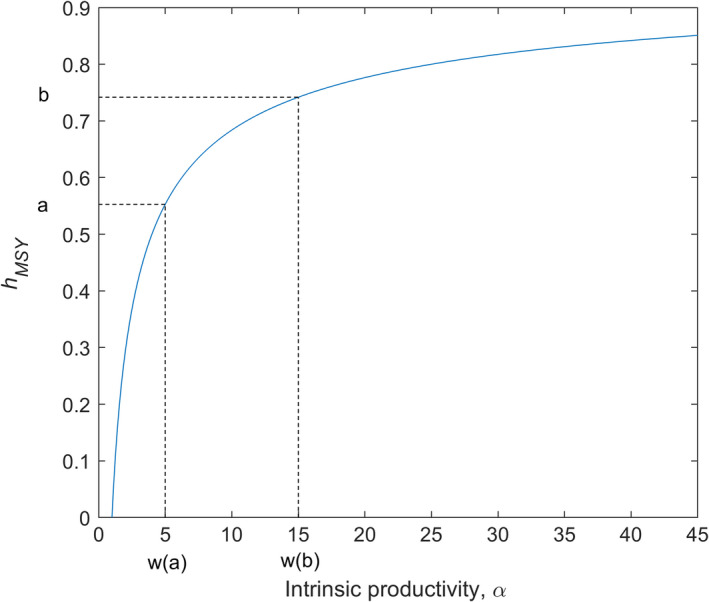
The harvest rate associated with maximum sustained yield (*y*‐axis) is an increasing function of intrinsic productivity (*x*‐axis). The transformation‐of‐variables technique is needed to convert a distribution of uncertainty in the x‐axis into a corresponding distribution on the scale of the *y*‐axis

## Data Availability

This paper does not use empirical data.

## APPENDIX A

If uncertainty in intrinsic productivity, α, is modeled with a lognormal distribution and the harvest rate associated with maximum sustained yield is

$$h_{\text{MSY}}=1‐\sqrt{\frac{1}{\alpha }},$$

then what is the density function for $h_{\text{MSY}}$? It may be tempting to simply replot the density $f\left(\alpha \right)$ over the transformed variable $h_{\text{MSY}}$ = $u\left(\alpha \right)$ = $1‐\sqrt{\frac{1}{\alpha }}$. However, this is incorrect because rescaling the *x*‐axis changes the area over which an integrand must equal 1 to satisfy the law of total probability. The change‐of‐variables technique is needed.

The change‐of‐variables technique stipulates that

$$g\left(h_{\text{MSY}}\right)=f\left[w\left(h_{\text{MSY}}\right)\right]\cdot \left|w^{\prime }\left(h_{\text{MSY}}\right)\right|.$$

Let $f\left(\alpha \right)$ be the lognormal distribution modeling uncertainty in intrinsic productivity. Since $h_{\text{MSY}}$ = $u\left(\alpha \right)$ = $1‐\sqrt{\frac{1}{\alpha }}$ is differentiable and always increasing over the range of *α* (Figure A1), the inverse function, *w*, can be found such that *α* = *w*(*h*_MSY_):

$$w\left(h_{\text{MSY}}\right)=\alpha =\frac{1}{{\left(h_{\text{MSY}}‐1\right)}^{2}}.$$

Further, the derivative of *w* (*h*_MSY_), $w^{\prime }\left(h_{\text{MSY}}\right)$, can be readily found:

$$w^{\prime }\left(h_{\text{MSY}}\right)=\frac{d}{dh_{\text{MSY}}}\left(\frac{1}{{\left(h_{\text{MSY}}‐1\right)}^{2}}\right)=‐\frac{2}{{\left(h_{\text{MSY}‐1}\right)}^{3}}.$$

Thus, we obtain the density function for *h*_MSY_:

$$g\left(h_{\text{MSY}}\right)=\int_{‐\infty }^{\infty }\frac{1}{\frac{1}{{\left(h_{\text{MSY}}‐1\right)}^{2}}\sigma \sqrt{2\pi }}e^{\left(‐\frac{{\left(\ln\left(\frac{1}{{\left(h_{\text{MSY}}‐1\right)}^{2}}s\right)‐\mu \right)}^{2}}{2\sigma^{2}}\right)}\left|‐\frac{2}{{\left(h_{\text{MSY}}‐1\right)}^{3}}\right|,$$

which is plotted in Figure A2 for *μ* = 1.75 and *σ* = 0.5.

Consulting Figure A1, note that

$$\begin{array}{cc} P\left[a<h_{\text{MSY}}<b\right] & =P\left[w(a)<\alpha <w(b)\right]\\ & =\int_{w(a)}^{w(b)}f\left(\alpha \right)d\alpha \\ & =\int_{a}^{b}f\left[w\left(h_{\text{MSY}}\right)\right]w^{\prime }\left(h_{\text{MSY}}\right)dh_{\text{MSY}}. \end{array}$$

Using numerical techniques, it is easy to compute $\int_{5}^{15}Log\;Normal\left(\alpha ,\;1.75,\;0.5\right)d\alpha =0.583$. The theory used in this Appendix is confirmed by computing the equivalence:

$$\int_{1‐\sqrt{\frac{1}{5}}}^{1‐\sqrt{\frac{1}{15}}}Log\;Normal\left(\frac{1}{{\left(h_{\text{MSY}}‐1\right)}^{2}},\;1.75,\;0.5\right)‐\frac{2}{{\left(h_{\text{MSY}}‐1\right)}^{3}}dh_{\text{MSY}}=0.583.$$

Appendix S3 contains MATLAB code supporting the ideas developed in this Appendix.
